# Maternal consumption of green tea extract during pregnancy and lactation alters offspring's metabolism in rats

**DOI:** 10.1371/journal.pone.0199969

**Published:** 2018-07-18

**Authors:** Ana C. L. Hachul, Valter T. Boldarine, Nelson I. P. Neto, Mayara F. Moreno, Eliane B. Ribeiro, Claudia M. O. do Nascimento, Lila M. Oyama

**Affiliations:** Universidade Federal de São Paulo, Escola Paulista de Medicina, Departamento de Fisiologia, São Paulo, Brasil; East Tennessee State University, UNITED STATES

## Abstract

**Introduction:**

Green tea extract has anti-inflammatory and antioxidant effects which improve dyslipidemia and decrease adipose tissue depots associated with hyperlipidic diet consumption.

**Objective:**

To evaluate the effect of green tea extract consumption by rats during pregnancy and lactation on the metabolism of their offspring that received control or high-fat diet with water during 10 weeks after weaning.

**Methods:**

Wistar rats received water (W) or green tea extract diluted in water (G) (400 mg/kg body weight/day), and control diet (10 animals in W and G groups) during pregnancy and lactation. After weaning, offspring received water and a control (CW) or a high-fat diet (HW), for 10 weeks. One week before the end of treatment, oral glucose tolerance test was performed. The animals were euthanized and the samples were collected for biochemical, hormonal and antioxidant enzymes activity analyses. In addition, IL-10, TNF-α, IL-6, and IL-1β were quantified by ELISA while p-NF-κBp50 was analyzed by Western Blotting. Repeated Measures ANOVA, followed by Tukey's test were used to find differences between data (p < 0.05).

**Results:**

The consumption of high-fat diet by rats for 10 weeks after weaning promoted hyperglycemia and hyperinsulinemia, and increased fat depots. The ingestion of a high-fat diet by the offspring of mothers who consumed green tea extract during pregnancy and lactation decreased the inflammatory cytokines in adipose tissue, while the ingestion of a control diet increased the same cytokines.

**Conclusion:**

Our results demonstrate that prenatal consumption of green tea associated with consumption of high-fat diet by offspring after weaning prevented inflammation. However, maternal consumption of the green tea extract induced a proinflammatory status in the adipose tissue of the adult offspring that received the control diet after weaning.

## Introduction

Maternal nutrition during intrauterine development influences the metabolism in fetuses and newborns, by exerting epigenetic modifications that can change the phenotype affecting the development of the fetus, this is defined as metabolic programming [1–3].

Green tea is derived from the plant *Camellia sinensis*, which is rich in polyphenols, and among them catechins, such as epigallocatechin, epicatechin, epicatechin gallate, and epigallocatechin-3-gallate, the latter being the most abundant [4–6].

These bioactive components have shown antioxidative roles [4, 7–10], they also lower fat depots and body mass [4, 8–10], increase fat oxidation [4, 8], improve insulin activity [4, 7], increase energy expenditure, and upregulate metabolism [8].

Consumption of a hyperlipidic diet, rich in saturated fatty acids, increases endotoxemia and contributes to systemic inflammation inducing the secretion of proinflammatory cytokines by activation of TLR-4 [11–13]. For example, in a study with mice that consumed hyperlipidic diet, it was shown that the animals developed insulin resistance accompanied by increased circulating endotoxin and gene expression of IL-6, TNF-α, IL-1β, and PAI-1 in visceral and subcutaneous adipose tissue deposits [14].

Consumption of green tea extract by mice fed with hyperlipidic diet leads to a decrease in the gain of body fat mass [15]; reduction in IL-1β, TNF-α, and LPS in liver [16]; and reduced weight gain with improved insulin resistance [17]. This suggests that green tea intake can prevent the alterations promoted by a hyperlipidic diet in adipose tissues and gut. These studies strongly indicate a beneficial effect of green tea consumption on the alterations promoted by a hyperlipidic diet.

The aim of this study was to evaluate the effect of green tea extract consumption associated with control diet by rats during pregnancy and lactation on the metabolism of their adult offspring receiving either control or high-fat diet with water for 10 weeks after weaning.

## Materials and methods

### Animals and treatments

The Ethics Committee on the Use of Animals of the Universidade Federal de São Paulo approved all procedures for the care of the animals used in this study, following international recognized guidelines (CEUA n°: 718008/2013). The rats were kept under controlled conditions of light (12-h light/ 12-h dark cycle with lights on at 07:00) and temperature (24 ± 1°C).

Three-month-old female Wistar rats (10 animals per group) were left overnight to mate, and copulation was verified the following morning by the presence of sperm in vaginal smears.

On the first day of pregnancy, the dams were isolated in individual cages and randomly divided into two groups: water (W) and green tea extract diluted in water (G), and both received control diet. The treatment was maintained throughout pregnancy and lactation. In the G group the water was completely substituted by green tea extract solution.

On the day of delivery, considered day 0 of lactation, litter sizes were adjusted to nine offspring each. The offspring weight was recorded weekly.

After weaning one male offspring from each mother was allocated in the following groups: WCW–offspring from mothers who received water and continued receiving control diet and water; GCW–offspring from mothers who received green tea extract and received control diet and water; WHW–offspring from mothers who received water and received high-fat diet and water; and GHW—offspring from mothers who received green tea extract and received high-fat diet and water, for 10 weeks.

The mothers and offspring (28d-old) not used after weaning were euthanized. The mothers were healthy and the adult offspring did not receive green tea extract. Data about the mothers and offspring in the end of lactation (28d-old) are presented in the article: “Effect of the consumption of green tea extract during pregnancy and lactation on metabolism of mothers and 28d-old offspring.” with DOI: 10.1038/s41598-018-20174-x.

The green tea extract, courtesy of Finlay Tea Solutions UK Ltd, was offered in an amber bottle daily at the concentration of 400mg/kg body weight/day diluted in the water according to the volume ingested in the previous day. The composition of green tea according to the manufacturer's certificate of analysis contained 4.98% caffeine and 39.17% polyphenols. The quantification of catechins of the green tea extract used in this study was performed by HPLC and the components identification was as it follows: 16 μg/mg catechin, 29 μg/mg epicatechin, 24 μg/mg epicatechin gallate, 40 μg/mg epigallocatechin gallate and 58 μg/mg epigallocatechin.

The control and high-fat diets were adapted according to the recommendations of the American Institute of Nutrition (AIN-93) [18].The growth diet was offered during pregnancy, lactation and offspring until 60d-old, period when the protein and mineral requirements are higher, and the maintenance diet was offer for offspring from 60 d-old until the end of treatment. The composition of the diet is presented in Table 1.

**Table 1:** Composition of the diet according to AIN-93 diet (g/kg).

Nutrients	Control diet—Growth (g/kg)	Control diet—Maintenance (g/kg)	High-fat diet—Growth (g/kg)	High-fat diet—Maintenance (g/kg)
Carbohydrates (g)	629.5^#^	720.7^#^	550^##^	600^###^
Carbohydrates (kcal)	63.8%	75.8%	42.5%	47.1%
Protein (g)	200	140	250	180
Protein (kcal)	20.3%	14.7%	19.3%	14.1%
Lipids (g)	70*	40*	220**	220**
Lipids (kcal)	16%	9.5%	38.2%	38.8%
Fiber (g)	50	50	0	0
Vitaminmix (g)	10	10	10	10
Mineral mix (g)	35	35	35	35
L-Cysteine (g)	3	1.8	3	1.8
Cholinebitartrate (g)	2.5	2.5	2.5	2.5
Tert-butylhydroquinone (g)	0.014	0.008	0.014	0.008
Energy value	3.9kcal/g	3.8kcal/g	5.2kcal/g	5.1kcal/g

# only cornstarch

## 450g cornstarch and 100g sugar

### 450g cornstarch and 150g sugar

* only soybean oil

** 40g soybean oil and 180g lard

### Oral Glucose Tolerance Test (OGTT)

At one week before the end of treatment, all animals were fasted for 12 hours. Initially, the baseline blood was collected to assess basal glucose concentration from the tail vein. Then a glucose solution (1.4 g/kg of body weight) was administrated by gavage. Blood samples were collected again after 15, 30, 45, 60 and 120 minutes to obtain the glycemic curve. The Homeostasis Model Assessment Insulin Resistance (HOMA-IR) was calculated taking into consideration fasting insulin (μU/mL) and fasting glucose (mmol/L), as follows: HOMA-IR = (insulin × glucose)/22.5.

### Experimental procedures

At the end of the experimental period (10 weeks after lactation's end), rats were euthanized by decapitation after 12h of fasting. Trunk blood was collected and immediately centrifuged (1258g, 15 minutes, 4°C). The serum was separated and stored at -80°C for later analyses. Retroperitoneal (RET), mesenteric (MES) and gonadal (GON) white adipose tissue, gastrocnemius muscle (GAST) and liver were isolated, weighed, immediately frozen in liquid nitrogen and stored at -80°C. The index of adiposity was calculated by the sum of MES, GON, and RET adipose tissue relative weight. To calculate the delta of body weight, we used the following formula: final weight minus initial weight.

### Biochemical and hormonal serum analyses

The serum cholesterol, HDL-cholesterol and triacylglycerol concentrations were measured using a commercial enzymatic colorimetric kit (Labtest^®^, Brazil, catalog number: 76; 13 and 87, respectively). Insulin (Millipore^®^, USA, EZRMI-13K), leptin (Millipore^®^, USA, EZRL-83K), lipopolysaccharideo (LPS) (Lonza^®^, QCL-1000) and adiponectin (AdipoGenLife Sciences^®^, AG-45A-0005) concentrations were quantified using specific commercial kits. Analyses were performed according to the manufacturer’s instructions.

### Antioxidant enzymes activity

The liver was weighted and homogenized in phosphate buffer. Superoxide dismutase (SOD) and glutathione peroxidase (GPx) enzyme activities in the serum were determined using RANSOD (SD125) and RANSEL (RS504) Kits (Randox Laboratories, Crumlin, UK), respectively, and analyzed accordingly to manufacturer's instructions. Catalase activity was measured by hydrogen peroxide consumption method [19]. The protein concentration in liver was measured by the Bradford method [20].

### Tissue total protein extraction

Total proteins from the tissues were extracted for ELISA and Western Blotting protocols.

For this, following decapitation, samples of the RET, MES and GON adipose tissue (0.3 g), GAST (0.15 g) and liver (0.1 g, all taken from the same lobe) were homogenized in 800μL of chilled extraction buffer (100mM Trizma Base pH7.5; 10mM EDTA;100mM NaF; 10mMN A_4_P_2_O_7_; 10mMN A_3_VO_4_; 2mM PMSF; 0.1mg/ml aprotinin). After homogenization, 80μl of 10% TritonX-100 was added to each sample. These samples were kept on ice for 30 minutes and then centrifuged (20817 g, 40minutes, 4°C).The supernatant was saved, and protein concentrations were determined using the Bradford assay (Bio-Rad, Hercules, California) with bovine serum albumin as a reference.

### IL-10, TNF-α, IL-6, and IL-1β protein concentration determined by ELISA

The quantitative assessment of IL-10, TNF-α,IL-6 and IL-1β proteins was carried out in total protein extract of RET, MES and GON adipose tissue, GAST and liver using ELISA (DuoSet ELISA, R&D Systems, Minneapolis, MN, USA) following the recommendations of the manufacturer.

### Protein analysis by Western Blotting

Total protein extract of GON and MES adipose tissue and liver were denatured by boiling (5 min) in a Laemmli sample buffer containing 100 mM DTT. Proteins from adipose tissue (30μg) and liver (75 μg) were separated using 10% sodium dodecyl sulfate (SDS) polyacrylamide gel electrophoresis in a Bio-Rad miniature slab gel apparatus. The electrotransfer of proteins from gels to nitrocellulose membranes was performed for ~1.30 h/4gels at 15 V (constant) in a Bio-Rad semi-dry transfer apparatus, in transfer buffer containing methanol (20%) and SDS (0.02%). Nonspecific protein binding to the nitrocellulose was reduced by preincubation for 2 h at 22°C in blocking buffer (wash buffer: Tris–HCl, 0.01M; NaCl, 0.15M; Tween 20, 0.02% and bovine serum albumin (BSA), 1%) for 2 h at 22°C. The membranes were rinsed thoroughly with wash buffer and incubated with primary antibodies (1:1000) overnight at 4°C in blocking buffer. The membranes were washed 3 times for 10min and incubated with horseradish peroxidase-conjugated secondary antibodies (1:5000) for 1 h at room temperature, then rinsed 3 times for 10min. Chemiluminescence (Thermo Fisher Scientific, Waltham, MA, USA) were visualized in a gel documentation system (Alliance 4.7, UVitec, Cambridge, UK). For evaluation of protein loading, membranes were stripped and reblotted with a standardized anti-beta-tubulin antibody. Band intensities were calculated with Scion Image (Scion Corporation 4.0.3.2). The following primary antibodies were purchased from Santa Cruz Biotechnology (Dallas, TX, USA): p-NF-κB p50 (sc-33022). Anti-β-tubulin (#2146) was purchased from Cell Signaling Technology (Danvers, MA, USA). Secondary antibodies were purchased from Sigma-Aldrich (St. Louis, MO, USA).

### Statistical analysis

All results were presented as means ± standard error of the mean (SEM). The statistical significance of the differences between the means of the samples of the groups was assessed using Repeated Measures ANOVA, followed by Tukey's test. Differences were considered to be significant when p < 0.05. This analysis compares control diet versus high-fat diet, and interaction between diet and treatment, considering treatment as the consumption of the green tea extract by the mothers.

## Results

### Body weight, delta and body weight gain

**Fig 1:**
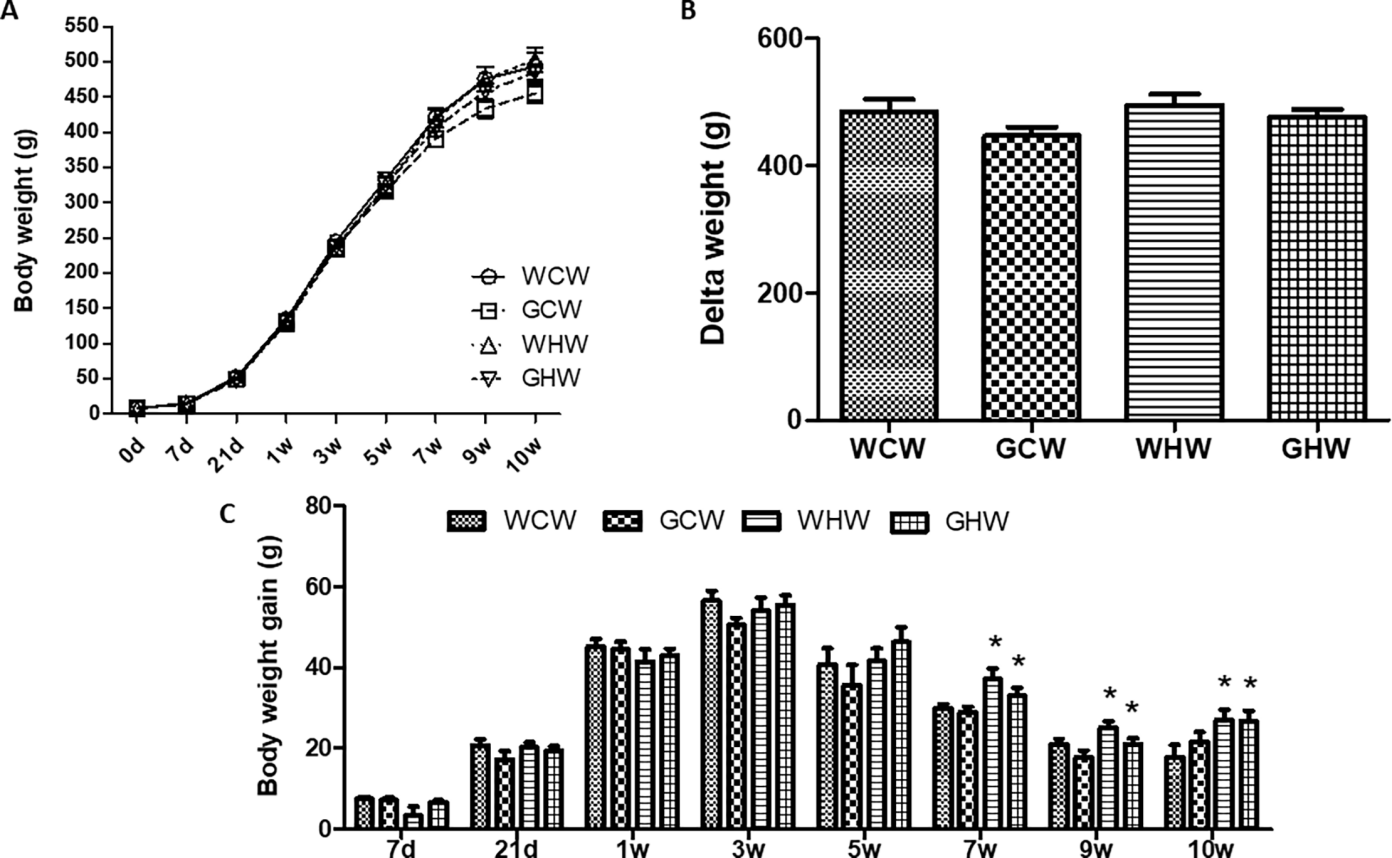
**Body weight evolution in the adult offspring: (A) Body weight; (B) Delta weight and (C) Body weight gain.** Data are mean ± standard error of the means (SEM) (n = 9–10). *p < 0.05 control diet versus high-fat diet. WCW–mother control diet and water and offspring control diet and water; GCW–mother control diet and green tea extract and offspring control diet and water; WHW–mother control diet and water and offspring high-fat diet and water; GHW–mother control diet and green tea extract and offspring high-fat diet and water. 0d -21d represent the lactation period and 1w-10w represent the period of treatment the offspring’s after weaning.

Adult offspring showed no difference in body mass at birth and at the end of lactation; similar observations were made in mothers regardless of the treatment with green tea during pregnancy and lactation. Treatments with high-fat diet did not affect the adult offspring final weight or delta weight at 10 weeks of treatment. However, at weeks 7, 9, and 10, adult offspring consuming high-fat diet had higher body weight gains than the control group (p < 0.01, p = 0.01, and p < 0.01, respectively) (Fig 1).

### Serum analyses

The consumption of a high-fat diet by the offspring for 10 weeks, independently of the mothers’ consumption of green tea, promoted increased insulin and leptin levels (p = 0.04; p < 0.01) along with an increase in HOMA-IR (p < 0.01) compared with on control diet (Table 2). The adiponectin/SAT ratio and triacylglycerol levels were lower in the high-fat diet than in the control diet (p = 0.02 and p < 0.01, respectively).

**Table 2:** Serum analysis cholesterol, HDL-cholesterol, triacylglycerol, insulin, adiponectin, leptin, LPS, adiponectin/SAT, and HOMA-IR.

	WCW	GCW	WHW	GHW	p value (diet)	p value (diet^v^treatment)
Cholesterol (mg/dL)	75.25±2.68	76.92±2.44^$^	84.13±7.59	66.98±2.03^$^	0.89	0.02^$^
HDL-cholesterol (mg/dL)	35.51±2.60	44.06±2.68^$^	36.69±2.24	34.41±1.85^$^	0.09	0.03^$^
Triacylglycerol (mg/dL)	237.10±27.66	209.49±18.00	175.42±23.41*	145.32±7.19*	<0.01*	0.94
Insulin (ng/mL)	2.80±0.73	3.59±0.76	4.59±0.98*	4.05±0.62*	0.04*	0.21
Adiponectin (μg/mL)	1.75±0.10	1.84±0.15	1.82±0.07	1.67±0.18	0.74	0.46
Leptin (ng/mL)	13.84±3.37	12.36±1.72	24.95±2.17*	20.12±1.57*	<0.01*	0.45
LPS (EU/mL)	10.07±1.12	7.22±0.53^$^	7.78±0.84	9.18±0.79^$^	0.81	<0.01^$^
Adiponectin/SAT	0.06±0.01	0.06±0.00	0.05±0.00*	0.04±0.00*	0.02*	0.67
HOMA-IR	16.86±5.06	24.01±6.07	32.54±7.18*	34.03±5.00*	<0.01*	0.49

Data are means ± SEM (n = 5–10).

*p < 0.05 control diet versus high-fat diet

^$^p < 0.05 interaction between diet^v^treatment.

Diet: control or high-fat; Treatment: consumption of the green tea extract by the mothers. WCW–mother control diet and water and offspring control diet and water; GCW–mother control diet and green tea extract and offspring control diet and water; WHW–mother control diet and water and offspring high-fat diet and water; GHW–mother control diet and green tea extract and offspring high-fat diet and water.

The consumption of the green tea extract by mothers decreased the total cholesterol in the GHW group compared with those in the WHW group (p = 0.02); and increased the HDL-cholesterol in the GCW group compared with those in the WCW group (p = 0.03). LPS was lower in the GCW group than in the WCW group; and higher in the GHW group than in the WHW group (p = 0.02 and p < 0.01, respectively) (Table 2).

### Oral Glucose Tolerance Test (OGTT)

**Fig 2:**
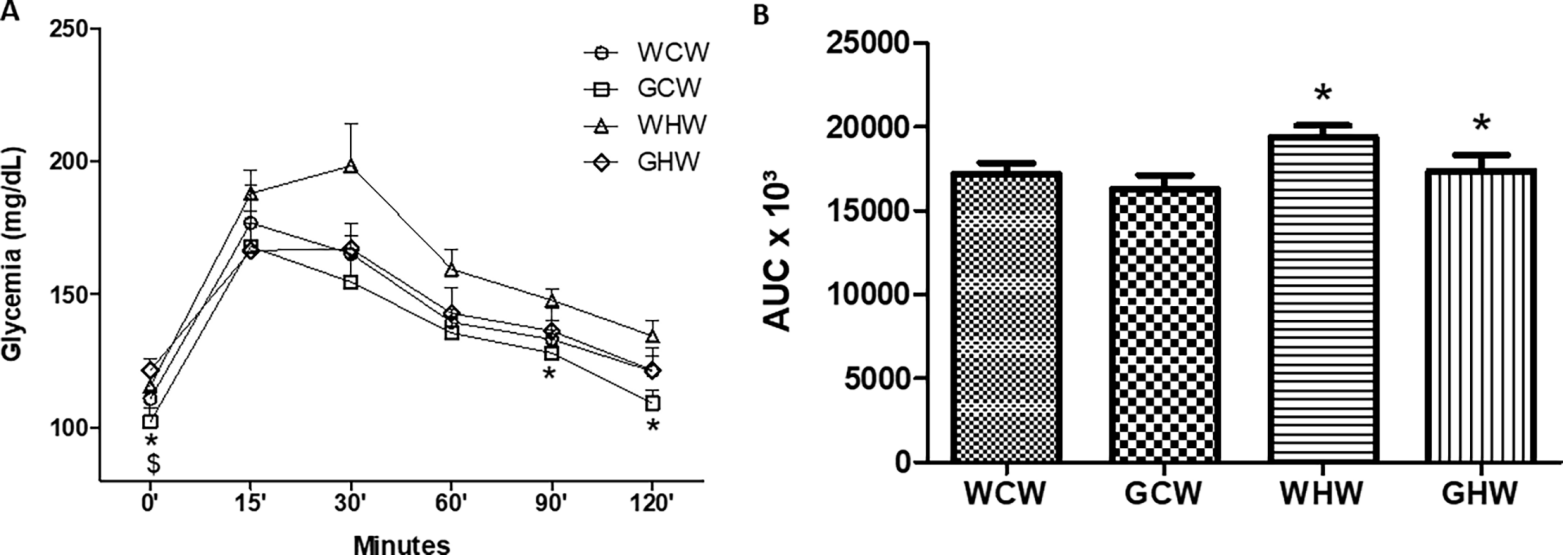
**Oral Glucose Tolerance Test (OGTT) in the experimental groups: (A) Oral Glucose Tolerance Test evaluation and (B) Area Under the Curve from OGTT.** Data are means ± SEM (n = 7).*p < 0.05 control diet versus high-fat diet;^$^p < 0.05 interaction between diet^v^treatment. Diet: control or high-fat; Treatment: consumption of the green tea extract by the mothers. WCW–mother control diet and water and offspring control diet and water; GCW–mother control diet and green tea extract and offspring control diet and water; WHW–mother control diet and water and offspring high-fat diet and water; GHW–mother control diet and green tea extract and offspring high-fat diet and water.

The glucose tolerance, as measured by the OGTT, differed significantly between the high-fat and the control groups after 0, 90, and 120 minutes (p < 0.01, p = 0.04, and p = 0.04). The AUC between the high-fat groups and the control groups also differed significantly (p = 0.03) (Fig 2). The adult offspring of the mothers who consumed the green tea extract exhibited reduced basal glycemia when associated with control diet and increased basal glycemia when associated with high-fat diet according to the results of OGTTs (p = 0.02).

### Relative tissue weight

The maternal consumption of green tea did not promote differences in the relative weight of the tissues of their adult offspring. The relative GON, MES, and SAT weights were significantly higher in rats consuming high-fat diets as compared to those in rats consuming control diets (p < 0.01), but there were no differences in other relative weights among groups (Table 3).

**Table 3:** Relative tissue weight of the retroperitoneal, gonadal and mesenteric adipose tissues, liver, gastrocnemius muscle, and sum of adipose tissues.

(g tissue/100g body weight)	WCW	GCW	WHW	GHW	p value (diet)	p value (diet^v^treatment)
RET	3.08±0.37	2.76±0.25	3.26±0.26	3.39±0.13	0.06	0.28
GON	2.47±0.32	2.27±0.16	2.83±0.21*	2.88±0.18*	0.01*	0.51
MES	1.26±0.11	1.07±0.08	1.59±0.14*	1.52±0.08*	<0.01*	0.44
LIVER	2.38±0.17	3.2±0.10	3.25±0.06	2.97±0.10	0.25	0.37
GAST	0.90±0.12	0.84±0.02	0.78±0.02	0.82±0.01	0.32	0.44
SAT	6.82±0.75	6.11±0.45	7.69±0.57*	7.80±0.20*	<0.01*	0.28

Data are means ± SEM (n = 9–10).

*p < 0.05 control diet versus high-fat diet.

RET–retroperitoneal adipose tissue; GON–gonadal adipose tissue; MES–mesenteric adipose tissue; GAST–gastrocnemius muscle and SAT–sum of adipose tissue. WCW–mother control diet and water and offspring control diet and water; GCW–mother control diet and green tea extract and offspring control diet and water; WHW–mother control diet and water and offspring high-fat diet and water; GHW–mother control diet and green tea extract and offspring high-fat diet and water.

### Antioxidant enzyme activities

The values for liver SOD, GPx, and catalase activities did not change significantly among the different groups (Table 4).

**Table 4:** Antioxidant enzymes activities.

(units/mg protein)	WCW	GCW	WHW	GHW	p value (diet)	p value (diet^v^treatment)
SOD	818.27±217.53	506.64±232.84	598.62±153.90	594.97±80.61	0.72	0.42
GPx	0.45±0.15	0.28±0.14	0.44±0.16	0.43±0.11	0.98	0.92
Catalase	362.31±114.69	174.55±104.42	212.20±64.71	193.25±27.42	0.30	0.75

Data are means ± SEM (n = 4–6). p < 0.05 was considered significantly different. SOD—superoxide dismutase and GPx—glutathione peroxidase. WCW–mother control diet and water and offspring control diet and water; GCW–mother control diet and green tea extract and offspring control diet and water; WHW–mother control diet and water and offspring high-fat diet and water; GHW–mother control diet and green tea extract and offspring high-fat diet and water.

### Tissue cytokine content

**Fig 3:**
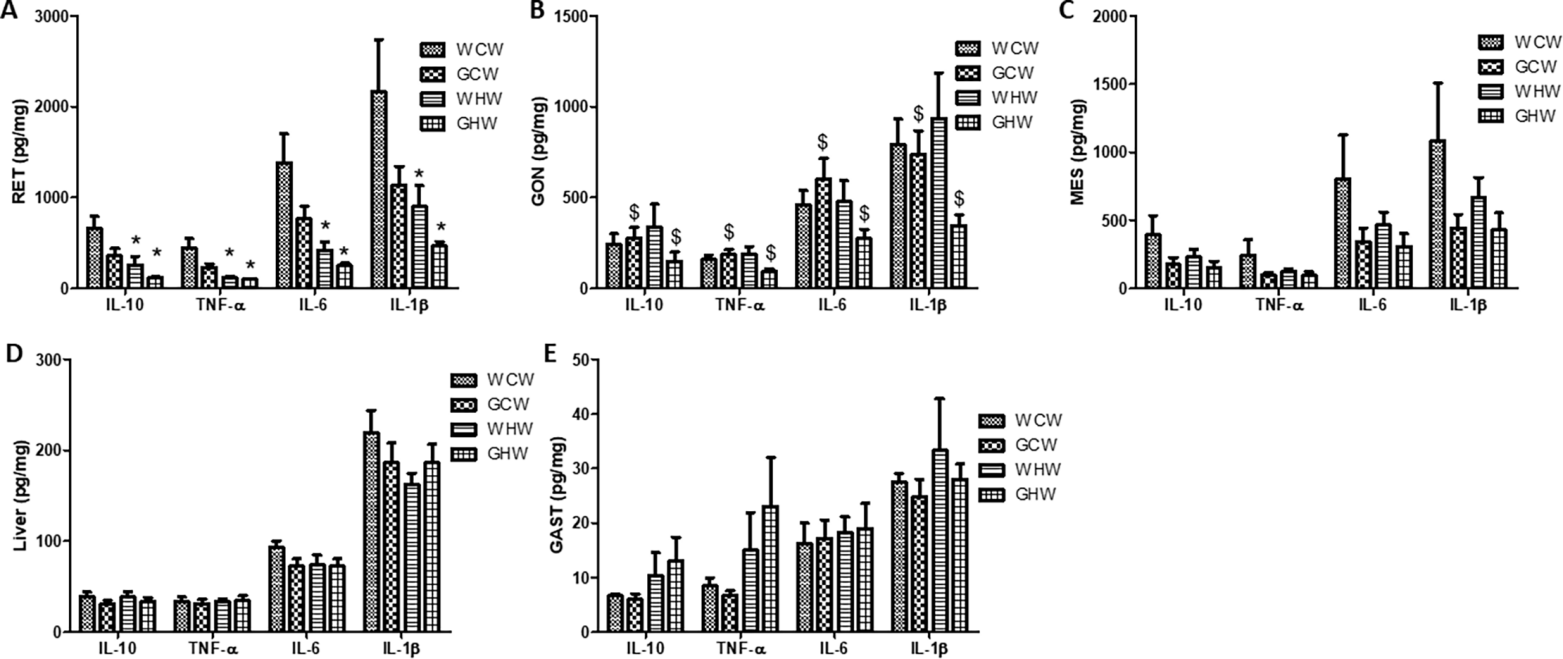
Tissues cytokine content. **(A) IL-10, TNF-α, IL-6 and IL-1β in RET; (B) IL-10, TNF-α, IL-6 and IL-1β in GON; (C) IL-10, TNF-α, IL-6 and IL-1β in MES; (D) IL-10, TNF-α, IL-6 and IL-1β in Liver and (E) IL-10, TNF-α, IL-6 and IL-1β in GAST.** Data are means ± SEM (n = 8–10).*p < 0.05 control diet versus high-fat diet;^$^p < 0.05 interaction between diet^v^treatment. Diet: control or high-fat; Treatment: consumption of the green tea extract by the mothers. RET–retroperitoneal adipose tissue; GON–gonadal adipose tissue; MES–mesenteric adipose tissue and GAST–gastrocnemius muscle. WCW–mother control diet and water and offspring control diet and water; GCW–mother control diet and green tea extract and offspring control diet and water; WHW–mother control diet and water and offspring high-fat diet and water; GHW–mother control diet and green tea extract and offspring high-fat diet and water.

Fig 3A shows that the RET levels for IL-10, IL-6, TNF-α, and IL-1β were lower in the adult offspring that received the high-fat diet compared to the adult offspring that received the control diet (p < 0.01, p < 0.01, p < 0.01, and p = 0.01, respectively).

The GON levels of IL-10, IL-6, TNF-α, and IL-1β were affected by consumption of the green tea extract during pregnancy and lactation, promoting a decrease in the GHW group (p = 0.02, p = 0.04, p = 0.03, and p = 0.04, respectively). However, IL-10, IL-6, and TNF-α were increased and IL-1 β was decreased in the GCW group relative to the levels in other groups (Fig 3B).

The IL-10, TNF-α, IL-6 and IL-1β of the MES, liver, and GAST did not differ among the groups (Fig 3C, 3D and 3E).

However, the IL-10/TNF-α ratio was not significantly different among the groups in any of the tissues analyzed (data not shown).

### Quantification of inflammatory proteins

**Fig 4:**
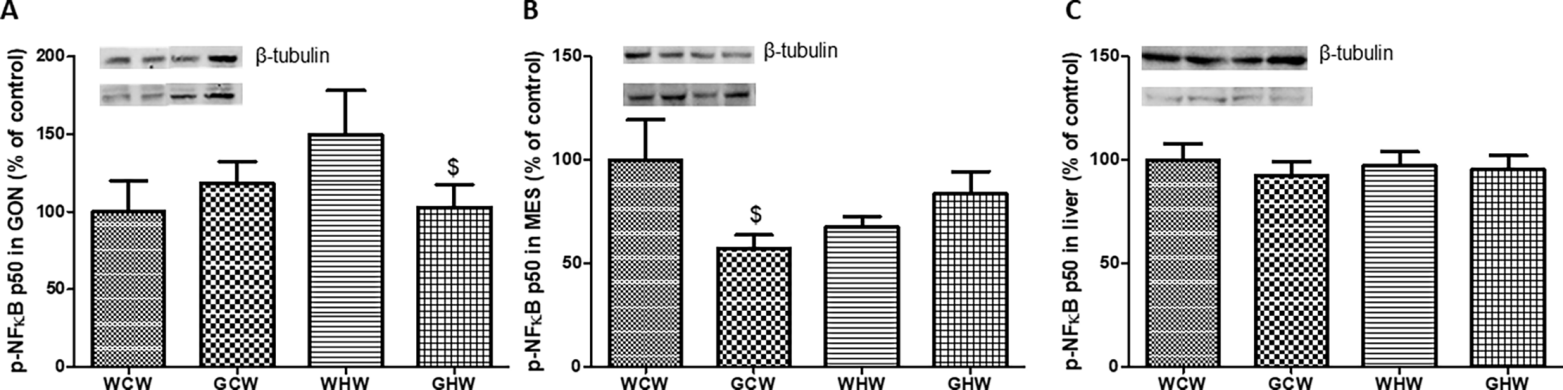
**Quantification of p-NF-kB p50 protein levels in the experimental groups: (A) Gonadal Adipose Tissue (GON); (B) Mesenteric Adipose Tissue (MES) and (C) LIVER. Molecular weight of p-NF-κB p50: 50kDa and molecular weight of β-tubulin: 55kDa.** Data are means ± SEM (n = 6–7).^$^p < 0.05 interaction between diet^v^treatment. Diet: control or high-fat; Treatment: consumption of the green tea extract by the mothers. WCW–mother control diet and water and offspring control diet and water; GCW–mother control diet and green tea extract and offspring control diet and water; WHW–mother control diet and water and offspring high-fat diet and water; GHW–mother control diet and green tea extract and offspring high-fat diet and water.

Western blotting analyses showed that the p-NF-κB p50 content in MES adipose tissue was lower in the GCW group (p = 0.02) than in the WCW group, and it was lower in the gonadal adipose tissue of the GHW group (p = 0.03) than that of the WHW group (Fig 4).

## Discussion

The consumption of a high-fat diet by rats for 10 weeks after weaning promoted alteration in glucose metabolism, fat depots, and inflammatory cytokines in the adipose tissue. The ingestion of a high-fat or control diet by the offspring of mothers who consumed green tea extract during pregnancy and lactation caused a modification in the inflammatory cytokines and p-NF-κB p50 in adipose tissues of their adult offspring.

Consumption of the high-fat diet for 10 weeks after weaning was effective in promoting obesity in the animals, impairing the glucose control and insulin responsiveness, and elevating the proinflammatory status. This can be observed by an increase in insulin, leptin, HOMA-IR, adipose tissue deposits, and sum of adipose tissue, and a decrease in adiponectin/SAT, in agreement with other studies, in mice or obese humans[21–23]. Additionally, several studies showed that the consumption of a high-fat diet increased the index of adiposity resulting in increased adipocyte size and the recruitment of macrophages, which could lead in an increase in the level of proinflammatory cytokines in white adipose tissue [24, 25].

The triacylglycerol levels were lower in the high-fat group than in the control group. Our control diet was rich in carbohydrates than the high-fat diet, because this macronutrient was modified in the high-fat diet for alteration of lipid concentration. The literature since 1960s has shown that consumption of carbohydrates, especially sugar, leads to increased lipids concentration in serum [26–29].

Additionally, the consumption of a high-fat diet after weaning led to a decrease in cytokines (IL-10, IL-6, TNF-α, and IL-1β) in retroperitoneal adipose tissue. According to the literature, the adipose tissue in obesity is characterized by increased production and secretion of inflammatory molecules, such as TNF-α and IL-6, which may have local and systemic effects [30–32]. A study with Wistar rats that received high-fat diet for 66 days and were euthanized at different times throughout a 24-h cycle showed the daily alterations of plasma adiponectin, IL-1, IL-6, and TNF-α in the two groups of animals (control diet and high-fat diet). Moreover, it has been demonstrated that the normal daily pattern of plasma concentrations of cytokines became disrupted in obese rats. The authors concluded that a high-fat diet causes insulin resistance and signs of inflammation, thereby disrupting the daily pattern of several hormones and adipokines, promoting significant effect on the circadian organization of neuroendocrine and immune responses [33]. Another study showed that a high-fat diet can influence biological clocks genes in adipose tissue providing information on the metabolic implications [34]. Thus, it is possible that the alterations in inflammatory cytokines could be affected by the circadian cycle.

On the other hand, studies show that the ingestion of high-fat diet altered the composition of the intestinal microbiota by increasing *Firmicutes*-to-*Bacteroidetes* ratio. As a reminder, the *Firmicutes* phylus are gram-negative and the *Bacteroidetes* phylus are gram-positive [35–37].

Additionally, the ingestion of a high-fat diet is associated with loss of the intestinal barrier and increased intestinal permeability to LPS, a component of the external cellular membrane of gram-negative bacteria, by disruption of tight cell junctions [38, 39]. The elevation of LPS concentration in plasma has a potent immuno-stimulatory effect in the host by inducing toll-like receptor 4 (TLR4) activation in the cell membranes and contributes to systemic inflammation by inducing the secretion of proinflammatory cytokines, such as IL-6 and TNF-α [11, 12, 39, 40]. Similar to LPS, saturated fatty acids are also recognized by membrane receptors that trigger proinflammatory-signaling pathways [41, 42].

Therefore, these data demonstrate that our high-fat diet model after weaning was able the promote obesity, and alterations in glucose metabolism and in the tissue inflammatory milieu.

The central aim of our study was to test the hypothesis that the ingestion of green tea by the mothers, during the pregnancy and lactation, might protect their offspring in adulthood from adverse effects of a high-fat diet. The literature showed that the ingestion of green tea concomitant with the high-fat diet protective effect of on glucose metabolism and the inflammatory process [16, 17, 22, 43–45].

Sato, M [46], studying rats, showed that the ingestion of low-protein diet during pregnancy, associated with green tea extract in control diet during lactation and standard diet after weaning upregulates AMPK activation and modulates this metabolic signaling cascade in the kidneys of adult male offspring. A study with mice demonstrated that maternal consumption of a high-fat diet supplemented with resveratrol during pregnancy and lactation was able to improve insulin sensitivity and reduce obesity in the offspring receiving the high-fat diet [47]. Furthermore, the rat offspring exposed to fetal malnutrition during pregnancy associated with ingestion of azuki bean polyphenol during lactation exhibited upregulation of AMPK phosphorylation in the liver and in skeletal muscle in the adults offspring [48].

The ingestion of green tea by the mothers did not prevent the effects of high-fat consumption by the adult offspring on the glucose metabolism, but it decreased the basal glucose levels in the adult offspring that received the control diet. Previous studies have reported the improvement of glucose tolerance by consumption of green tea associated with high-fat diet [22, 44]. This suggests that green tea may affect metabolic programming, but the effects of high-fat diet overlaps the effect of green tea on glycemic metabolism.

The adult offspring from green tea consuming mothers who received high-fat diet had lower total cholesterol levels, but those ingesting a control diet had high HDL-cholesterol levels. It is clear from the literature that a high-fat diet alters the total cholesterol content, and the improvement of the lipid profile by the green tea has been demonstrated by Maron, Lu [49]. Total cholesterol and LDL-C levels were lowered and HDL-cholesterol was increased with a mix containing green tea catechins in capsules given daily for 12 weeks to individuals who are on a habitual Chinese diet. Batista, Cunha [50] offered 250 mg of dry green tea extract in the form of capsules for 8 weeks and observed reduction in total cholesterol and no difference in HDL-cholesterol, in humans.

In our study, the maternal consumption of green tea extract associated with the ingestion of high-fat diet by the offspring after weaning promoted a decrease in total cholesterol accompanied by a reduction in inflammatory cytokines in gonadal adipose tissue.

As stated by Cunha, Lira [45] mice treated with a high-fat diet associated with green tea extract (400 mg/kg body mass/day), showed increased HDL-cholesterol without alteration in their levels of total cholesterol and decreased TNF-α in mesenteric adipose tissues. Mice treated with a control diet and green tea extract had increased IL-10 levels in mesenteric adipose tissues (proving beneficial effects of the green tea consumption) [45]. It is important to emphasize that in our study only the mothers consumed green tea, as opposed to the studies which offered green tea associated with high-fat diet in other life periods.

Our results demonstrate that green tea ingestion during pregnancy and lactation has a potential effect on the lipid profile, and may promote changes in the inflammatory response. It is important to emphasize the importance of being careful with the nutritional strategies adopted during pregnancy and lactation, since they may influence fetal development by altering metabolic programming, resulting in alterations in the metabolic responses in adulthood dependent of the diet.

The maternal consumption of green tea in association with the offspring high-fat diet ingestion decreases IL-10, IL-6, TNF-α, and IL-1β, and p-NF-κB p50 levels in gonadal adipose tissue, although it has increased LPS levels. As of now, it is important to emphasize that physiologically the differences in LPS concentrations do not appear to influence the inflammatory process in the adult offspring, despite the statistical differences.

According to the literature, the treatment of obesity by EGCG is linked with the suppressor of the TLR/NFκB pathway. The mechanism involves the binding of EGCG to the 67-kDa laminin receptor, which induces the Tollip (toll interacting protein) signaling pathway, a negative regulator of TLR4, thereby attenuating LPS-mediated inflammation by suppressing the TNF-α and IL-1β levels in macrophages, leading to the downregulation of inflammatory responses. Thus the effects of LPS are inhibited in a dose-dependent fashion by the EGCG pretreatment, which reduces the synthesis of the proinflammatory cytokines TNF-α, IL-1β, and IL-6, as well as nuclear translocation of NF-κB p50/p65 [51–53].

The anti-inflammatory effects of green tea have been attributed to its polyphenol content, and this suggests that the polyphenols are able to suppress chronic inflammation [54, 55]. Additionally, green tea reduces TLR4 levels, blocking proinflammatory effects. And, EGCG in immune cultured cells had an anti-inflammatory effect, which was partially explained by inhibition of the TLR [56].

An unexpected result was the negative effect of the consumption of the green tea extract, during pregnancy and lactation, which promoted a proinflammatory status in adipose tissue of adult offspring that consumed control diet. This was confirmed by an increase in the cytokines IL-6, TNF-α, and IL-10 associated with a decrease in IL-1β in gonadal adipose tissue, despite the decrease in p-NF-κB p50 levels in mesenteric adipose tissue and in LPS levels. However, it seems that the proinflammatory milieu in the adipose tissue was not able to modify carbohydrate metabolism given that no glucose intolerance was detected.

Similarly, the maternal consumption of grape seed procyanidins and control diet seems to cause a cardiovascular disease-prone phenotype in their adult offspring [57]. These studies demonstrated the effects of polyphenols in metabolic programming.

## Conclusion

In conclusion, our results showed that obesity, glucose intolerance, and possibly insulin resistance were induced by the consumption of a high-fat diet for 10 weeks. The maternal consumption of the green tea extract had a protective role against dyslipidemia, glucose intolerance, and accumulation of adipose tissue in the adult offspring that received the high-fat diet after weaning. Nonetheless, maternal consumption of the green tea extract induced a proinflammatory milieu in the adipose tissue of the adult offspring that received control diet after weaning showing that consumption of green tea extract is capable of altering the metabolic development of offspring by modifying metabolic programming. More studies are required to better understand the mechanism underlying this effect, and to further elucidate the role of green tea extract ingestion during pregnancy and lactation on the adult offspring metabolism programming.

## Supporting information

S1 File Data—Fig 1. Body weight evolution. (PDF)

S2 File Data—Table 2. Serum analyses. (PDF)

S3 File Data—Fig 2. OGTT. (PDF)

S4 File Data—Table 3. Relative tissue weight. (PDF)

S5 File Data—Table 4. Antioxidant enzymes activities. (PDF)

S6 File Data—Fig 3. Tissue cytokine content. (PDF)

S7 File Data—Fig 4. Quantification of inflammatory proteins. (PDF)
