# Supplementary material for: Maternal consumption of green tea extract during pregnancy and lactation alters offspring's metabolism in rats
Source: PLoS One. 2018 Jul 18;13(7):e0199969. doi: 10.1371/journal.pone.0199969 (PMC6051583; doi:10.1371/journal.pone.0199969)
Supplement: S1 File — (PDF) [file pone.0199969.s001.pdf]

Groups	Body weight evolution																	
	Body weight (g)									Delta weight (g)	Body weight gain (g)							
	0d	7d	21d	1w	3w	5w	7w	9w	10w		7d	21d	1w	3w	5w	7w	9w	10w
WCW	8.12	15.28	46.43	118.18	202.75	284.3	354.11	395.34	406.8	398.68	7.16	15.93	40.03	46.03	34.7	28.22	19.04	11.46
WCW	6.96	11.52	36.91	115.33	215.7	306.99	384.65	436.3	446.7	439.74	4.56	15.11	45.31	48.77	40.52	34.84	22.1	10.4
WCW	7.5	15.7	57.44	142.96	251.32	344	408.2	447.2	460.55	453.05	8.2	25.1	49.98	51.36	42.8	28.96	15.8	13.35
WCW	8.68	15.65	50.95	146.33	264.62	328.9	419.35	477.21	485.91	477.23	6.97	18.64	39.26	60.04	25.2	26.53	20.91	8.7
WCW	8.03	14.87	49.41	133.24	240.27	300.1	397.96	447.47	458.61	450.58	6.84	18.31	38.11	56.51	21.6	25.79	20.64	11.14
WCW	9.9	19.77	61.16	158.42	281.4	377.8	474.38	533.07	559.24	549.34	9.87	25.13	49.05	64.72	52.2	31.61	18.74	26.17
WCW	8.44	16.46	59.5	149.93	271.83	364.5	458.38	516.02	534.95	526.51	8.02	23.75	50.81	64	42.7	32.6	23.48	18.93
WCW	6.43	13.76	46.96	133.15	237.28	329.1	417.38	471.72	498	491.57	7.33	17.82	50.17	51.32	46.8	29.92	19.05	26.28
WCW	8.57	17.03	62.67	116.14	240.29	362.7	476.72	558.88	593.4	584.83	8.46	27.08	45.25	65.51	60	31.07	29.62	34.52
GCW	8.37	13.73	50.6	140.5	249.66	354.28	427.1	472.8	481.7	473.33	5.36	19.87	52.32	56.7	45.25	33.15	20.6	8.9
GCW	6.59	13.3	44.95	149.38	252.27	317.3	383.33	417.42	430.7	424.11	6.71	18.99	41.08	49.04	30.5	24.58	13.49	13.28
GCW	7.46	13.88	47.76	138.8	242.26	314.3	375.69	408.25	428.47	421.01	6.42	20.7	46.62	45.24	38.7	29.91	12.46	20.22
GCW	8.2	15.8	45.15	146.45	262.85	333	415.44	468.15	501.06	492.86	7.6	14.42	44.36	58.98	42.8	33.65	25.04	32.91
GCW	6.48	11.78	43.31	138.58	247.21	333.2	415.43	458.2	483.92	477.44	5.3	16.82	47.06	56.43	45.1	32.23	20.78	25.72
GCW	8.92	19.73	64.15	157.57	270.23	363.4	445.61	504.8	530.66	521.74	10.81	0	52.47	52.08	43.9	28.1	23.01	25.86
GCW	8.72	17.2	54.69	129.15	228.01	311.7	379.61	414.13	430.26	421.54	8.48	24.41	46.49	47.17	36	22.72	13.19	16.13
GCW	7.53	15.52	48.2	114.82	203.73	273.4	353.76	388.44	414.7	407.17	7.99	21.59	38.8	44.9	-6.2	28.47	10.36	26.26
GCW	8.37	16.32	48.13	109.46	212.21	295.5	379.62	429.15	459.11	450.74	7.95	15.86	42.48	51.14	44.2	33.24	15.43	29.96
GCW	7.26	13	48.9	94.57	187.52	265.7	332.34	374.47	391.25	383.99	5.74	19.53	33.43	44.07	37.4	23.52	23.63	16.78
WHW	7.69	14.22	52.67	129.07	203.56	290.81	360.76	402.65	420.1	412.41	6.53	24.57	42.65	36.89	36.16	27.4	22.12	17.45
WHW	8.11	14.62	53.56	150.9	269.03	373.75	474.4	542.1	557.8	549.69	6.51	20.5	54.77	58.22	39.86	50.9	34.9	15.7
WHW	8.26	13.43	43.65	117.42	219.76	309.5	395.37	445.1	469.8	461.54	5.17	17.43	39.83	49.41	39.4	48.14	26.7	24.7
WHW	7.4	14.06	44.31	150.3	271.35	361.1	462.58	528.53	557.37	549.97	6.66	16.76	44.1	62.27	44.3	35.11	24.1	28.84
WHW	6.15	11.15	39.78	114.33	206.5	264.3	345.51	407.28	432.35	426.2	-6.15	16	36.94	44.01	23.7	35.73	23.69	25.07
WHW	10.2	20.28	63.92	146.75	247.01	326.6	412.18	465.27	495.65	485.45	-10.2	25.71	42.17	48.49	38.8	29.7	23.68	30.38
WHW	7.65	14.83	54.47	142.69	267.63	365.5	468.33	517.77	554.01	546.36	7.18	22.5	48.5	64.8	46.6	39.75	18.5	36.24
WHW	6.42	14.06	46.21	120.24	234.53	337.6	437.68	499.25	528.65	522.23	7.64	17.22	43.06	58.34	55.9	32.83	24.28	29.4
WHW	7.07	14.51	54.22	80.39	189.97	282.8	393.46	474.13	510.54	503.47	7.44	22.01	21.45	63.96	50.1	35.14	28.41	36.41
GHW	8.03	13.72	46.97	133.55	252.73	359.03	442.7	492	507.4	499.37	5.69	17.67	50.12	61.38	42.93	46.02	24.7	15.4
GHW	7.47	15.28	50.84	165.35	286.05	360.7	437.22	478.43	500.5	493.03	7.81	21.9	46.89	62.54	40.5	27.68	16.08	22.07
GHW	6.54	11.05	38.95	114.22	198.92	301.2	332.91	389.96	412.97	406.43	4.51	17.11	39.05	42.19	76.6	28.85	26.99	23.01
GHW	7.91	14.65	47.98	150.35	269.32	351.4	453.37	517.36	553.59	545.68	6.74	18.65	36.45	61.01	48.7	37.47	24.22	36.23
GHW	5.98	9.99	41.19	134.97	245.1	324.9	415.78	457.84	487.43	481.45	4.01	16.84	36.61	61.69	44.4	33.53	13.84	29.59
GHW	8.79	18.29	62.41	152.09	262.72	335.9	416.06	472.48	496.05	487.26	9.5	25.21	48.14	53.58	33.4	29.44	26.38	23.57
GHW	8.56	15.49	51.25	129.15	233.6	322.5	402.46	450.03	475.53	466.97	6.93	22.31	47.7	56.79	39.6	24.7	19.47	25.5
GHW	7,34	13,82	50,7	118,48	229,92	319,7	414,47	472,3	492,49	485,15	6,48	20,96	44,17	59,05	43,3	36,46	18,24	20,19
GHW	8,24	15,55	42,84	99,13	187,33	273,6	359,93	407,14	448,55	440,31	7,31	14,71	40,7	44,2	45,8	35,39	17,27	41,41
GHW	7,28	15,06	49,58	103,73	206,48	294,4	383,5	441,94	473,01	465,73	7,78	19,72	40,99	52,56	47,9	31,72	23,07	31,07
