# Supplementary material for: Maternal consumption of green tea extract during pregnancy and lactation alters offspring's metabolism in rats
Source: PLoS One. 2018 Jul 18;13(7):e0199969. doi: 10.1371/journal.pone.0199969 (PMC6051583; doi:10.1371/journal.pone.0199969)
Supplement: S2 File — (PDF) [file pone.0199969.s002.pdf]

Groups	Serum analyses									
	Cholesterol (mg/dL)	HDL-cholesterol (mg/dL)	Triacylglycerol (mg/dL)	Glycemia (mg/dL)	Insulin (ng/mL)	Adiponectin (μg/mL)	Leptin (ng/mL)	LPS (EU/mL)	Adiponectin/SAT	HOMA-IR
WCW	71.6	31.02	126.03	103	0.933	1.834	5.285	11.46	0.13	5.685
WCW	91.72	26.93	233.88	119	1.926	1.49	11.638	10.96	0.06	13.56
WCW	78.99	54.64	294.63	124	0.492	1.566		10.92	0.06	3.612
WCW	65	34	139.22	108	1.105	1.5	8.127	10.847	0.06	7.058
WCW	69	34	173.01	105	2.871	2.102	9.128	8.736	0.08	17.832
WCW	76	31	300.13	114	5.293	1.65	21.168	7.28	0.03	35.691
WCW	71	37	210.34	102	5.739	2.156	16.954	7.899	0.05	34.626
WCW	82	36	375.28		5.797		5.378	17.218		
WCW	72	35	281.46		1.13		33.104	5.387		
GCW	79.29	32.68	257.85	125	3.324	1.676	19.384	10.07	0.05	24.579
GCW	80	37	294.07	108	6.122	1.796	17.667	7.5	0.06	39.113
GCW	73	40	174.02		1.966	1.62	7.129	6.84	0.08	
GCW	86	56	178.06		3.752	2.502	11.86	6.84	0.08	
GCW	70	37	250.69	112	3.704	1.518	13.553	6.479	0.05	24.539
GCW	90	58	278.44	85	5.906	2.336	17.57	5.46	0.05	29.694
GCW	66	41	130.64	97	0.373	1.45	4.929	7.389	0.08	2.14
GCW	72	49	169.48	100			12.173			
GCW	82	49	208.32	88			15.435			
GCW	71	41	153.34				3.991			
WHW	66.27	31.63	92.98	116	2.459	1.844	14.623	9.07	0.07	16.873
WHW	100.59	31.02	126.45	125	3.464	1.54	22.583	9.44	0.03	25.613
WHW	131.36	30.59	91.32	120	0.871	1.722		9.87	0.09	6.18
WHW	71	46	193.19	112	8.217	1.684	32.962	12.376	0.04	54.437
WHW	74	36	176.54	109	4.796	2.118	20.683	6.625	0.07	30.924
WHW	76	44	249.18	112	5.272	1.96	25.143	4.951	0.04	34.928
WHW	103	46	205.3	115	8.651	1.896	29.404	6.043	0.04	58.851
WHW	74	31	300.13		6.902		31.585	6.771		
WHW	61	34	143.76		0.703		22.645	4.878		
GHW	61.83	25.19	146.28	135	4.129	1.89	27.522	11.7	0.05	32.969
GHW	56	25	133.67	130	6.734	1.418	19.281	8.117	0.03	51.78
GHW	77	41	197.23		2.375	1.676	20.262	10.374	0.05	
GHW	62	32	131.65		2.098	1.356	24.859	5.242	0.03	
GHW	68	38	144.26	117	3.416	1.414	15.916	10.556	0.04	23.64
GHW	63	34	131.15	112	5.468	1.25	13.219	8.773	0.04	36.228
GHW	72	41	133.17	103	4.198	2.694	17.785	9.501	0.08	25.578
GHW	74	40	170,49	121			27,793			
GHW	70	34	119,55	133			17,825			
GHW	66	34	145,78				16,802			
