# Supplementary material for: Maternal consumption of green tea extract during pregnancy and lactation alters offspring's metabolism in rats
Source: PLoS One. 2018 Jul 18;13(7):e0199969. doi: 10.1371/journal.pone.0199969 (PMC6051583; doi:10.1371/journal.pone.0199969)
Supplement: S3 File — (PDF) [file pone.0199969.s003.pdf]

Groups	OGTT						
	Glycemia (mg/dL) - minutes						AUC x 10^3
	0'	15'	30'	60'	90'	120'	
WCW	103	243	189	138	114	105	17805
WCW	119	173	143	136	133	120	16575
WCW	124	210	188	162	165	148	20340
WCW	108	168	169	142	149	137	17917.5
WCW	105	149	160	136	132	117	16417.5
WCW	114	157	161	143	123	112	16492.5
WCW	102	138	147	120	116	109	14857.5
WCW							
WCW							
GCW	125	238	169	161	151	113	19365
GCW	108	146	160	132	145	123	16755
GCW							
GCW							
GCW	112	170	158	139	114	102	16065
GCW	85	125	125	117	116	106	13905
GCW	97	174	172	146	117	104	16657.5
GCW	100	155	116	112	96	90	13275
GCW	88	168	183	142	157	126	18157.5
GCW							
WHW	116	182	167	150	143	139	18232.5
WHW	125	193	285	169	170	153	22710
WHW	120	188	164	142	143	125	17835
WHW	112	228	206	197	137	105	20490
WHW	109	173	203	165	155	147	19785
WHW	112	152	166	151	143	133	17670
WHW	115	200	198	143	144	139	19012.5
WHW							
WHW							
GHW	135	199	190	191	175	148	21472.5
GHW	130	191	189	150	155	139	19327.5
GHW							
GHW							
GHW	117	175	139	119	120	111	15465
GHW	112	165	159	144	119	102	16312.5
GHW	103	128	128	114	114	110	14062.5
GHW	121	136	189	134	127	95	16455
GHW	133	172	176	149	144	146	18517.5
GHW							
