# Supplementary material for: Maternal consumption of green tea extract during pregnancy and lactation alters offspring's metabolism in rats
Source: PLoS One. 2018 Jul 18;13(7):e0199969. doi: 10.1371/journal.pone.0199969 (PMC6051583; doi:10.1371/journal.pone.0199969)
Supplement: S4 File — (PDF) [file pone.0199969.s004.pdf]

Groups	Relative tissue weight (g tissue/100g body weight)					
	RET	GON	MES	LIVER	GAST	SAT
WCW	2.31	1.003	0.849	3.275	0.862	3.349
WCW	2.509	1.918	1.077	4.294	0.865	5.505
WCW	2.58	2.37	0.92	3.45	0.72	5.87
WCW	2.37	1.46	1.24	3.19	1.88	5.07
WCW	2.21	2.32	0.94	2.55	0.87	5.48
WCW	4.77	3.7	1.75	3.13	0.73	10.23
WCW	3.55	3.22	1.48	2.82	0.8	8.24
WCW	4.73	2.44	1.48	3.81	0.69	8.65
WCW	3.55	3.81	1.64	3.01	0.69	9.01
GCW	3.52	2.52	1.26	3.36	0.86	7.3
GCW	3.32	2.77	1.24	3.61	0.79	7.33
GCW	2.25	1.71	0.98	3.22	0.9	4.95
GCW	3.08	1.97	1.42	3.19	0.86	6.48
GCW	2.45	2.46	1.12	3.78	0.72	6.04
GCW	4.17	3.07	1.25	3.02	0.73	8.49
GCW	1.91	1.58	0.55	3.12	0.98	4.04
GCW	3.02	2.09	1.04	2.85	0.81	6.15
GCW	2.27	2.89	1.07	3.1	0.83	6.23
GCW	1.62	1.71	0.79	2.75	0.93	4.12
WHW	2.31	1.95	1.11	3.21	0.81	5.38
WHW	3.62	3.3	1.52	3.14	0.87	8.44
WHW	2.28	1.6	0.9	3.02	0.9	4.79
WHW	2.51	2.87	1.55	3.36	0.72	6.93
WHW	3.31	2.8	1.35	3.53	0.79	7.45
WHW	4.39	3.13	1.88	3.2	0.81	9.39
WHW	3.44	3.04	1.95	3.03	0.68	8.42
WHW	4.31	3.29	2.13	3.51	0.7	9.73
WHW	3.2	3.56	1.98	3.27	0.77	8.74
GHW	3.5	2.64	1.48	3.12	0.75	7.62
GHW	3.53	3.83	1.46	3.55	0.81	8.82
GHW	3.84	2.64	1.56	2.87	0.83	8.04
GHW	3.4	2.7	1.68	2.71	0.8	7.78
GHW	3.42	3.02	1.22	3.13	0.8	7.66
GHW	2.44	3	1.23	3.15	0.87	6.67
GHW	3.88	1.95	1.47	2.79	0.87	7.3
GHW	3.27	3.42	1.62	3.24	0.82	8.32
GHW	3.69	2.19	1.38	2.46	0.9	7.26
GHW	3.01	3.42	2.15	2.74	0.8	8.58
