# Supplementary material for: Maternal consumption of green tea extract during pregnancy and lactation alters offspring's metabolism in rats
Source: PLoS One. 2018 Jul 18;13(7):e0199969. doi: 10.1371/journal.pone.0199969 (PMC6051583; doi:10.1371/journal.pone.0199969)
Supplement: S5 File — (PDF) [file pone.0199969.s005.pdf]

Groups	Antioxidant enzymes activities (units/mg protein)		
	SOD	GPx	Catalase
WCW	1656,535	0.869	604.723
WCW	766.777	0.348	288.035
WCW	679.077	0.175	178.908
WCW	572.208	0.222	151.234
WCW		0.992	812.054
WCW	416.779	0.15	138.943
WCW	1.656.535		
WCW			
WCW			
GCW	1551,668	0.963	686.641
GCW	442.327	0.185	89.396
GCW	665.498	0.353	138.667
GCW	345.985	0.149	104.021
GCW	8.363	0.02	9.724
GCW	26.011	0.025	18.861
GCW			
GCW			
GCW			
GCW			
WHW	461.503	0.33	207.045
WHW	635.648	0.155	107.579
WHW	245.326	0.181	102.011
WHW	489.178	1.238	524.807
WHW		0.296	149.6
WHW	1161,459	0.486	182.172
WHW			
WHW			
WHW			
GHW	837.477	0.533	219.935
GHW	612.5	0.345	238.361
GHW	316.914	0.155	66.664
GHW	768.15	0.703	171.057
GHW	608.268		244.603
GHW	426.541		218.907
GHW			
GHW			
GHW			
GHW			
