# Supplementary material for: Maternal consumption of green tea extract during pregnancy and lactation alters offspring's metabolism in rats
Source: PLoS One. 2018 Jul 18;13(7):e0199969. doi: 10.1371/journal.pone.0199969 (PMC6051583; doi:10.1371/journal.pone.0199969)
Supplement: S6 File — (PDF) [file pone.0199969.s006.pdf]

Groups	Tissue cytokine content																			
	RET (pg/mg)				GON (pg/mg)				MES (pg/mg)				LIVER (pg/mg)				GAST (pg/mg)			
	IL-10	TNF-α	IL-6	IL-1β	IL-10	TNF-α	IL-6	IL-1β	IL-10	TNF-α	IL-6	IL-1β	IL-10	TNF-α	IL-6	IL-1β	IL-10	TNF-α	IL-6	IL-1β
WCW	618	253.47	882.81	1500.38	323.02	87.8	360.6	729.1	402.37	107.57	641.08	916.84	31.55	25.69	96.24	199.64	7.2	4.2	25.95	
WCW	375.55	145.02	557.14	929.53	556.7	218.57	747.47	1542.26	396.76	139.26	609.06	894.5	38.02	22.51	81.85	155.26	6	6.16	35.42	29.19
WCW	621.42	243.27	1074.15	1667.22	407.49	177.19	667.18	1030.46	473.15	195.84	1025.45	940.12	23.38	20.57	70.21	173.1	5.59	5.78	25.31	30.33
WCW	341.12	329.2	696.08	1204.14	78.95	120.07	209.4	416.18	162.84	113.72	315.4	427.14	25.55	26.32	76.69	165.46	7.38	12.11	19.35	33.36
WCW	816.41	505.41	2013.95	3398.47	95.1	105.6	251.78	466.19	152.64	116.4	311.31	469.61	31.9	26.13	84.31	208.39	7.11	17.84	7.88	28.79
WCW	973.11	747.57	2040.88		195.36	200.43	553.92	803.81	138.02	103.55	289.56	485.52	65.49	60.93	138.83	397.17	5.44	4.78	0.83	18.79
WCW	592.2	495.25	1369.64	2365.47	186.58	186.53	437.37	847.88	259.26	167	503.73	775.02	38.05	26.57	85.74	195.25	5.08	7.07	6.17	27.63
WCW	186.47	153.77	463.36	673.1	51.46	53.52	146.43	127.88	94.6	78.7	247.66	369.57	61.08	53.94	116.05	272.28	7.36	9.57	14.31	23.04
WCW	1461.3	1111.85	3367.74	5568.72	300.76	283.67	763.81	1158.07	1459.08	1158.72	3287.7	4443.95	37.65	40.7	87.5	204.69	8.29	8.71	11.53	28.57
GCW	933.35	306.67	1472.73	2006.45	653.15	193.92	1101.88	1317.14	622.23	228.73	1191.16	1198.36	22.19	15.75	65.87	115.43	1.75	3.51	18.1	14.62
GCW	533.3	352.38	1169.24	1788.74	226.03	198.97	546.43	878.44	12.4	6.29	17.14	21.29	60.43	62.73	130.06	347.3	9.09	9.29	16.48	33.25
GCW	158.13	117.92	352.12	496.37	318.69	284	681.3	992.04	110.47	70.58	215.69	305.16	26.39	26.65	79.02	184.26	9.38	11.58	32.48	35.13
GCW	415.12	328.81	925.58	1390.77	260.29	217.9	357.66	743.69	166.72	123.82	344.45	526.53	35.97	34.57	73.46	195.69	7.72	7.02	15.7	27.82
GCW	100.32	85.44	240.25	361.91	285.95	211.47	996.03	924.42	165.98	110.34	330.58	493.57	43.53	42.76	86.72	245.61	2.26	3.61	2.22	15.43
GCW	359.11	304.46	896.12	1402.74	128.6	92.91	288.33	456.11	159.13	111.88	331.91	515.6	25.36	27.14	61.03	135.02	3.89	3.88	4.16	10.74
GCW	167.72	133.81	425.18	666.88	108.76	66.87	209.03	223.19	89.08	58	188.19	256.73	21.84	21.63	48.55	161.37	3.85	6.3	8.99	13.05
GCW	472.3	393.51	1294.46	1988.27	481.17	344.39	1080.1	1271.68	156.97	115.94	371.19	488.38	20.05	30.14	44.33	129.08	9.92	8.27	34.24	27.96
GCW	290.68	187.19	573.8	789.25	270.06	197.86	642.97	436.24	183.58	98.5	275.52	439.69	26.6	24.63	57.7	157.65	7.66	6.78	21.56	35.36
GCW	144.4	112.03	288.86	491.4	40.96	34.51	98.19	122.53	76.78	64.56	160.95	196.62	28.19	28.86	78.93	192.09	4.3	7.23	18.21	34.8
WHW	896.24	132.89	974.31	2057.8	906.86	241.99	1064.4	1914.4	586.49	165.64	1069.52	1261.82	75.53	31.79	144.39	197.89	3.57	1.98	15.79	
WHW	476.81	126.8	551.28	1545	1030.53	354.71	1075.19	2382.69	423.59	202.94	744.97	1487.38	59.13	24.71	82.71	152.09	3.63	2.2	19	14.54
WHW	231.35	63.24	311.45	839.93	401.42	114.63	425.85	1048.72	199.04	55.67	425.9	553.12	30.37	25.76	80.42	169.11	5.98	5.37	26.28	22.33
WHW	268.39	262.72	715.59	1153.74	47.5	44.19	96.2	220.73	71.16	70.29	170.27	227.18	32.54	42	76.31	168.16	8.78	13.9	23.88	35.72
WHW	140.5	138.5	362.86	652.2	127.83	128.48	289.03	596.81	125.85	106.71	271.21	377.47	27.68	33.41	49.23	139.76	12.1	11.04	12.55	23.46
WHW	73.99	77.71	215.31		185.27	426.59	455.12	946.88	215.87	171.16	424.73	599.89	23.7	28.11	42.31	121.56	6.8	8.19	17.58	30.36
WHW	87.98	109.17	245.27	344.1	103.57	125.82	264.5	504.01	237.09	182.22	539.89	766.65	39.11	49.19	79.16	222.79	4.27	16.49	16.11	20.8
WHW	90.83	81.14	241.43	367.97	127.28	149.77	365.03	486.51	98.25	73.78	216.12	322.79	36.67	41.53	74.56	181.98	4.33	6.17	0.67	20.96
WHW	76.07	50.29	176.58	254.58	109.52	97.11	244.71	319.42	127.51	95.35	308.55	423.24	20.46	26.99	34.36	111.29	43.45	69.23	31.77	97.8
GHW	150.36	62.64	230.68	687.62	575.98	141.78	588.44	182.39	227.86	63.01	528.69	672.65	21.26	11.56	76	129.35	2.41	2.24	18.83	20.98
GHW	161.92	98.59	337.75	579.25	40.33	43.62	109.76	226.21	56.79	31.56	114.51	150.48	60.91	74.45	113.93	310.66	41.14	83.47	33.96	47.76
GHW	65.24	49.05	118.06	236.78	62.88	52.05	121.3	203.07	189.46	166.52	414.4	581.95	35.65	41.51	87.94	201.86	36.67	67.88	50.75	30.16
GHW	129.48	117.8	267.59	417.43	119.04	125	271.96	497.56	44.65	31.56	103.24	104.93	24.74	29.83	75.61	151.91	10.81	17.67	24.17	31.61
GHW	67.5	55.19	136.59	219.76	42.89	43.9	107.71	214.44	49.56	38.14	93.5	136.49	49.38	42.77	103.97	268.31	8.3	14.3	17.83	26.15
GHW	121.29	108.1	277.45	461.18	133.28	127.82	336.3	517.66	29.33	18.25	47.14	85.22	36.09	29.51	59.99	183.5	9.57	12.73	14.36	34.42
GHW	132.31	174.52	415.17	619.82	125.99	110.45	331.89	435.73	497.11	321.96	979.12	1364.26	37.2	38.33	74.97	216.87	7.05	9.09	14.52	25.38
GHW	125.38	95.29	231.23	397.92	229.62	181.11	499.64	709.25	130.29	78.18	281.02	385.87	21.69	15.31	37.92	130.71	5.83	8.14	3.15	25.3
GHW	118.01	127.23	232.39	560.57	63.71	52.52	161.53	210.48	48.39	34.09	97.56	170.07	31.05	41.09	58.17	158.82	4.81	8.46	3.2	22.34
GHW	130.8	96.84	230.22	459.03	95.98	45.85	195.22	275.96	265.76	151.42	427.71	634.35	19.35	21.15	37.42	113.92	3.45	6.8	9.31	16.23
