# Supplementary material for: Maternal consumption of green tea extract during pregnancy and lactation alters offspring's metabolism in rats
Source: PLoS One. 2018 Jul 18;13(7):e0199969. doi: 10.1371/journal.pone.0199969 (PMC6051583; doi:10.1371/journal.pone.0199969)
Supplement: S7 File — (PDF) [file pone.0199969.s007.pdf]

Groups	Quantification of inflammatory proteins		
	p-NF-kB p50 in GON (% of control)	p-NF-kB p50 in MES (% of control)	p-NF-kB p50 in LIVER (% of control)
WCW	67.561	41.687	101.779
WCW	80.918	167.393	96.099
WCW	62.131	49.375	102.121
WCW	189.39	141.545	134.464
WCW	52.03	50.956	83.234
WCW	156.72	127.169	
WCW	91.25	121.875	82.302
WCW			
WCW			
GCW	94.838	71.242	111.036
GCW	104.851	58.225	105.607
GCW	137.9	61.057	104.802
GCW	118.271	69.854	81.152
GCW	117.494	63.386	77.372
GCW	68.666	56.622	74.977
GCW			
GCW	185.465	20.814	
GCW			
GCW			
WHW	91.626	70.064	112.574
WHW	129.38	70.018	104.538
WHW	132.861	66.168	86.335
WHW	210.124	39.98	109.529
WHW	62.285	79.37	101.508
WHW	284.202	77.547	
WHW	136.641	70.338	68.453
WHW			
WHW			
GHW	110.417	111.758	99.114
GHW	62.279	106.95	101.271
GHW	46.815	114.904	111.572
GHW	112.316	67.684	86.628
GHW	149.635	86.144	107.053
GHW	142.844	51.496	65.029
GHW			
GHW	95.333	44.867	
GHW			
GHW			
